# Incidental Finding of a Duplicated Vertebral Artery Followed by a Spinal Dural Arteriovenous Fistula on Spinal Angiography

**DOI:** 10.7759/cureus.11351

**Published:** 2020-11-05

**Authors:** Luke Mugge, Danielle D Dang, Brian P Curry, Michael Crimmins

**Affiliations:** 1 Neurological Surgery, Inova Neuroscience and Spine Institute, Falls Church, USA; 2 Neurological Surgery, Walter Reed National Military Medical Center, Bethesda, USA; 3 Neurology, Walter Reed National Military Medical Center, Bethesda, USA

**Keywords:** dual origin, aorta, subclavian, vertebral artery, dural arteriovenous fistula

## Abstract

Duplicated origin of the vertebral artery (VA) is an extremely rare normal anatomic variant. While most often considered non-pathological, duplicated origin carries an increased risk of dissection. An association with vascular pathologies such as aneurysms, arteriovenous malformations, and AV fistulas has been suggested. The objective is to describe this unusual anatomic variant with is concomitant vascular pathology and review current literature. The authors report a case of incidentally-discovered duplicated origin of the left VA in patients with a spinal dural arteriovenous fistula (dAVF). A 61-year-old man with a history significant for sarcoidosis presented with progressive lower extremity weakness and paresthesias. MRI of the thoracic spine demonstrated significant confluent edema and patchy contrast enhancement in the caudal spinal cord and conus medullaris which did not appear related to the patient's neurosarcoidosis. A diagnostic spinal angiogram incidentally demonstrated that the left V1 segment had a duplicated origin, one branch arising from the aortic arch and the other branch arising from the left subclavian artery, with union at the C5 transverse foramen. This finding represented an incidental anomaly discovery was noted to be incidental and was not believed to be related to the patients underlying pathology. Subsequently, a dAVF was discovered, originating from the right T7 spinal artery. Location of this vascular malformation directly correlated with the patient’s symptoms. The patient then underwent embolization of the spinal dAVF and recovered uneventfully. Duplicated origin of the VA is an extremely rare but well-described variant, most commonly involving the left VA. To our knowledge, this is the only reported spinal dAVF associated with duplicated origin of the left VA. An association with other pathological entities has been suggested, and thus this case adds to a growing body of cases characterizing these relationships.

## Introduction

Many variations have been described for the origin of the vertebral artery, yet the presence of a dual origin is not a common finding and is likely underrepresented in the literature [1]. Given the origin and course of the V1 segment can be highly diverse, its involvement in surrounding anatomy is not uncommon. This has significant surgical implications, precluding certain approaches or interventions [2,3]. From an embryology standpoint, the presence of a duplicated origin indicates a persistence of two segmental arteries has occurred and a local vascular malformation may be associated [4]. To our knowledge, this is the first described case of a duplicated origin of a V1 segment found in the same patient as a dural arteriovenous fistula (dAVF). 

## Case presentation

The patient is a male in his 60s with a past medical history significant for pulmonary sarcoidosis. He was referred to neurology clinic for progressive, bilateral, lower extremity numbness which he noted first in his feet. The patient indicated the pain and tingling in his feet worsened with prolonged standing and walking. He also endorsed lower back pain on follow-up visits. MRI of the thoracic cord demonstrated a longitudinally extensive transverse myelitis. The initial workup was unrevealing for an etiology and initial suspicions were that his condition was due to neurosarcoidosis. A lumbar puncture was performed and negative. Upon further review of the spinal imaging it was thought that there may be dilatation of the spinal epidural venous plexus, which would potentially indicate a dural arteriovenous fistula. The patient was then taken for a catheter angiogram for further evaluation.

**Figure 1:**
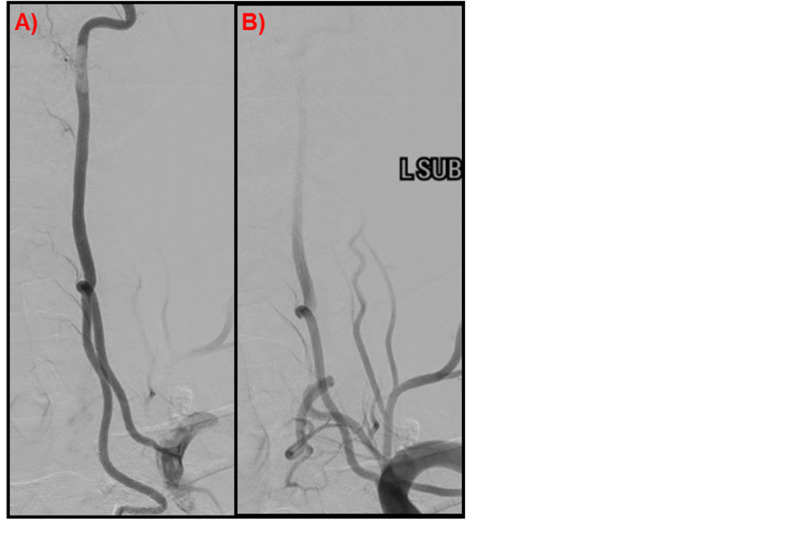
Left vertebral artery Spinal angiogram demonstrated duplicated origin of the left V1 Segment, (A) AP of the left vertebral artery; (B) V1 segment of the left vertebral artery, left subclavian injection.

During angiography, the left and right subclavian arteries were imaged to evaluate for spinal cord blood flow. An injection within the aorta was required to establish a road map for the left vertebral artery and the left vertebral artery origin from the aortic arch was found. An injection of the aortic arch origin of the vertebral artery revealed complete filling of all segments of the left vertebral artery. Retrograde filling of the left subclavian artery was seen indicating a potential dual origin from the left subclavian artery. A catheter was placed within the left subclavian and a second origin of the left vertebral artery was appreciated to originate and merge with the aortic V1 segment at the level of the C5 vertebrae (Figure 1). Equal filling was seen from both the aortic and subclavian origin. No aneurysms or dissections were appreciated in either origin or at the confluence. When the right T7 spinal artery was injected, a dAVF was observed. The fistula originated from a distal branch of the spinal artery and combined with an anterior spinal vein with abnormal filling appreciated. The rest of the spinal arteries were interrogated and noted to be grossly normal without aneurysms, fistulas, or other vascular abnormalities.

Post-procedure and follow-up

**Figure 2:**
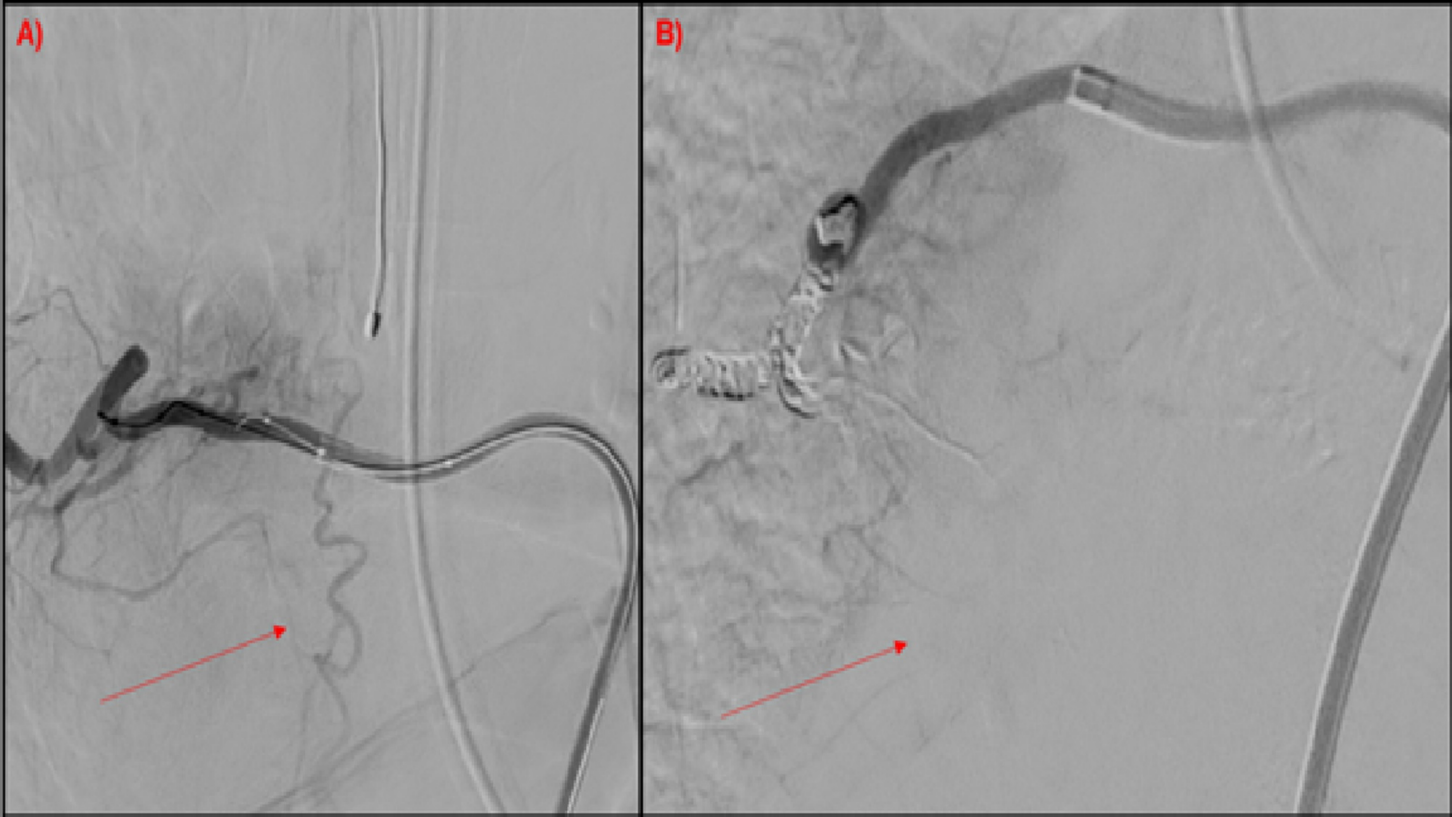
Spinal angiogram Spinal angiogram demonstrating successful embolization of the right T7 dAVF; (A) pre-embolization dAVF, (B) post-embolization with ablation of the dAVF. dAVF, dural arteriovenous fistula.

The patient underwent embolization of the right T7 spinal artery was performed with obliteration of the fistula (Figure 2). The patient tolerated the procedure well without complication and was discharged home.

## Discussion

Several published studies describe both left and right duplicate origin of the V1 segment [5]. Anatomically, the right vertebral origin branches have been described as both being from the subclavian, common carotid, and less commonly other blood vessels [6,7]. For the left side, origins have been described as both being from the aorta, both from the subclavian, and, as is the case for our study, from the aorta and the left subclavian [8-12]. By frequency, in cases where the left V1 was duplicated, 21 out of 27 cases had origins from the aorta and left subclavian [11]. This makes our specific anatomical variation among the most commonly seen when this specific anomaly is observed. While the majority of these cases were discovered incidentally, and some were seen in conjunction with local pathology, none have been reported to be found in conjunction with a dAVF. For our case, it is likely that the patient developed this dAVF as a result of the inflammation associated with his neurosarcoidosis. However, the presence of a duplicated origin demonstrates that this patient's vascular anatomy was aberrant in at least one location in a significant way. It is tempting to hypothesize that a similar anomalous vascular anatomical variation in the area of the spine predisposed this patient to the formation of a dAVF when under similar circumstances in an otherwise healthy patient, no such development would have occurred. 

Reviewing the embryologic development of the vertebral artery is crucial for understanding this variant. Early on, small intersegmental branch arteries extend from the dorsal aorta to supply the developing somites [13]. There are a total of seven intersegmental arteries within the cervical region and each anastomoses with the other. As development continues, the 7th intersegmental artery develops into the subclavian artery on that side giving rise to a proximal connection while the remaining more distal 6 intersegment arteries regress, leaving the anastomotic segments in place to form the vertebral artery [14]. Variation in origin can result anywhere along the developing vertebral artery when any of the intersegmental vertebral arteries persist, allowing for a continued anastomosis [15]. As this related to In our case, an additional, more distal intersegmental artery persisted in addition to the 7th artery as a dual origin. Prior studies suggest that the left 4th or 5th intersegmental artery originate from the aorta and so either of these two intersegmental arteries could be the source of the variation seen in our case [16].

A duplicated origin is classically asymptomatic, although one case was reported with symptoms of headache and dizziness [17]. Pathologic vascular abnormalities have been associated with a duplicated artery. Local variations, abnormalities, or pathologies are likely related to the aberrant development of local anatomy associated with incomplete regression of rudimentary vascular connections. This was likely the case for our patient. As previously stated above, we hypothesize that our patient has multiple congenital vascular abnormalities, which, when combined with neurosarcoidosis, led to the formation of a dAVF. Therefore, it is reasonable to incur that if one abnormal vascular abnormality is observed in a patient with a concomitant inflammatory disease process, that a more detailed assessment of for other vascular lesions be undertaken. 

Dural arteriovenous fistulas are the most common vascular abnormality of the spine. Clinical presentation can be highly variable, making the diagnosis challenging [18]. It should be included in the differential when patients are hemiplegic or have signs of myelopathy; while dAVF is a diagnosis that should not be missed, it is often delayed by 12 to 24 months [19]. In terms of location, a study by Tsuruta et al. demonstrated that 83.7% of spinal dAVF are within the thoracolumbosacral region and have a non-hemorrhagic presentation [20]. Our patient similarly presented with bilateral lower extremity weakness of unknown cause meriting further work-up with spinal angiography. As in our case, the dual origin variant is typically found incidentally during angiography and did not directly cause the patient's symptomatology.

## Conclusions

This case illustrates the importance of understanding the presentation and clinical implications of a duplicated origin of the V1 segment and the concomitant, systemic vasculopathies that may be associated. We propose that the combination of neurosarcoidosis and multiple congenital vascular variants, as indicated by this dual origin, predisposed this patient to the formation of a dAVF. We seek to emphasize that, while this presentation is an incidental finding, this variant has implications for the treatment of associated aneurysms or dissections, requiring modified endovascular techniques to accommodate the aberrant vascular course. We were fortunate that both malformations were discovered on the same spinal angiogram, negating the need for further vascular workup. Additional cases and research are needed in order to further characterize these rare vascular anomalies and their relationship to inflammatory vasculopathies as well as to determine differences in periprocedural risk during the endovascular treatment of cerebrovascular disease.
